# Psychiatry trainee stressors in a postgraduate psychiatry training centre in India

**DOI:** 10.1192/bji.2018.25

**Published:** 2019-08

**Authors:** Bharathram Sathur Raghuraman, Manamohan Nataraj, Lakshmi Shiva

**Affiliations:** 1 Senior Resident Doctor, Department of Psychiatry, National Institute of Mental Health and Neurosciences, India. Email: drsrbharathram@gmail.com

**Keywords:** Education and training, stigma and discrimination, low- and middle-income countries

## Abstract

Stress and burnout are major issues affecting medical trainees, especially psychiatry trainees, throughout the world. Stress and burnout were studied using an online survey among psychiatry trainees of the National Institute of Mental Health and Neurosciences (NIMHANS) which is one of the oldest and largest training centers in India. Postgraduate training in academic institutions like NIMHANS, while offering excellent teaching experience, may impact the mental and physical health of trainees due to complex clinical challenges and academic pressure. Measures need to be taken to enhance trainee well-being by ensuring support from colleagues and seniors, allowing for an adequate work–life balance, introducing departmental level committees to address grievances and providing therapy and mentorship. Providing safe and non-stigmatizing spaces to seek help in workplace promotes whole-person growth and well being.

Medical students and trainees have higher levels of stress and burnout compared with the general population (Dyrbye & Shanafelt, 2016). Burnout and stress may lead to various mental health problems and hence need to be addressed.

Symptoms of stress may vary within the medical professions and psychiatry residents have been found to have higher job burden and stress than other healthcare workers (Zuardi *et al*, 2011). In a study conducted among 276 psychiatry residents, 69% (190) met the criteria for burnout and 17% screened positive for depression. Lack of work–life balance and feeling unappreciated were major contributors of burnout (Holmes *et al*, 2017). In a study involving Italian psychiatry residents, 16% had lifetime suicidal ideations (Ferrari *et al*, 2015). Some of the factors influencing burnout were the race/ethnicity of the residents, their primary language and cultural background. Therefore, it is recommended that at-risk residents should be provided with cultural-awareness workshops, language-assistance programmes as well as senior resident and faculty mentors (Afzal *et al*, 2010). Interestingly, stigma related to the psychiatric profession is another major factor contributing to stress in psychiatry residents. In a study done in Belgium, 75% of all trainee psychiatrists reported hearing humiliating remarks about the psychiatric profession more than once, and trainees who had been in training for a longer period of time experienced a significantly higher level of stigmatisation (Catthoor *et al*, 2014). This evidence motivated us to assess burnout and stress among our residents as well.

Our training institution, the National Institute of Mental Health and Neurosciences (NIMHANS), is a large academic and tertiary care centre. It has 550 beds, with several specialty clinics and a fairly busy out-patient and emergency service. The out-patient department sees close to 100 new patients daily and an average of 400 people attend the follow-up clinics and specialty services. Nearly 30 people attend the emergency psychiatry service daily. In addition to the out-patient services, in-patient services are also provided, with each junior resident doctor overseeing the care of five to six in-patients on average.

To study burnout and stress, we conducted an online survey among junior and senior resident doctors. As there is no questionnaire standardised to measure trainee stress in India, this was a pilot attempt at creating one. The questionnaire was based on a literature search and expert consensus. It contained ten questions targeting various domains like perceived current mental health status of self and peers, perceived stress at work, support from peers and seniors, roles and responsibilities in the training, scope for personal development, etc. For example, a question on perceived stigma was framed as ‘how much do you think stigma plays a role in disclosing mental health issues in the department?’ with four answers to choose from, as indicated in Fig. 1. The anonymity of responses was maintained through the online portal. The questionnaire was sent through the online portal to all 142 residents, along with three reminders. Around 62% of the residents responded to the questionnaire.

**Fig. 1:**
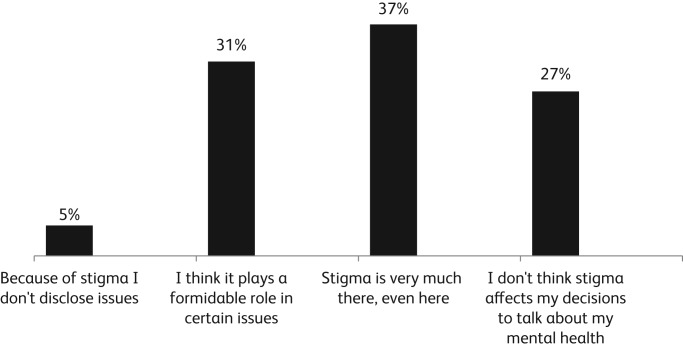
The level of perceived stigma among respondents to the online survey.

Responses showed that close to 71% of residents felt that they were able to manage the workload adequately. However, 72% felt that they did not feel comfortable disclosing their own personal mental health issues within the department due to concerns about stigma. Nearly half of residents rated their colleague's mental well-being as poor, whereas only around 24% rated their own only mental health as poor. An earlier study done in the same institution showed that personal supervision – including involvement of residents in leisure activities, social and administrative skills and help in liaison with other disciplines – resulted in lower satisfaction among junior residents than educational and clinical supervision. (Viswanath *et al*, 2013)

This survey was revealing as it highlighted how stigma prevented trainees from seeking help for mental health issues, even in a psychiatry department, which is ironic but concerning.

## Trainee life in NIMHANS – a personal account

In addition to managing the busy out-patient and in-patient services, each trainee resident is expected to participate in a fairly rigorous academic programme. There are daily teaching sessions which include a variety of teaching formats such as case presentations, academic seminars, journal clubs, objective structured clinical examination training, debates, faculty presentations and psychotherapy supervision (Chaturvedi *et al*, 2015). In addition, trainees undergo both formative and summative assessments and feedback in the form of ongoing 360° assessments every 6 months and formal exam-based assessments every 6 months.

Trainees that are already struggling to cope with these multiple responsibilities occasionally experience an incident with a patient or colleague that can result in stress or a mental health issue. Residents can be vulnerable and we have had a colleague take his own life during his final year of residency. This incident was extremely traumatic for the whole trainee group and healing took a long time. Additionally, residents may occasionally face bullying or additional personal and family stress.

In psychiatry, unpredictable incidents may also happen, for instance there was a patient from the forensic ward that fired a gun and took an entire ward hostage. This enhanced the sense of vulnerability among residents who already felt that they had to be vigilant when patients become aggressive or violent. The residency training, although well-organised, may put residents through a lot of other challenges which are not a part of the curriculum.

## What is being done to address stress among residents?

There are several ways in which departments and organisations can help psychiatry trainees to manage stress, such as to detect problems early and signpost colleagues to support services.

In our department, some measures that have been initiated include the following: a student welfare team has been formed which consists of representatives of junior residents, senior residents and consultants. This student-friendly committee has addressed various problems faced by residents, including work-related issues, bullying, personal stress and academic problems, and this has proven to be of timely help to many. A statutory committee, known as the Internal Complaints Committee, is in place specifically for addressing sexual harassment in the workplace. There is a suggestion/complaint box in the department for trainees to write about their problems anonymously, and these issues will be then addressed. A psychotherapy supervision programme held once every week provides scope for discussing personal stress. Senior residents run a formal peer mentorship programme for junior residents, which is another initiative to facilitate academic and personal guidance. In addition, specific remediation is provided by the faculty for students who seem to be lagging behind on their formal exam assessments. Feedback every 3 months, as part of the formative assessments, encourages trainees to discuss their concerns with their teachers. All of the above-mentioned measures have been in place in the department for the past 3 years and are being well used by the residents, however more structured studies are required to quantitatively and qualitatively analyse the effectiveness of such intervention programs. Studies in other centres indicate that whole-system healthy workplace interventions – like the interventions being implemented in NIMHANS – can improve health and well-being and can promote healthier behaviours in healthcare staff (Brand *et al*, 2017). An 8-week mentalisation-based stress reduction intervention for staff in a 12-bed mental health in-patient unit also resulted in a significant decrease in self-reported psychological distress, including reduced levels of self-reported anxiety, which indicates the need for individualised psychological interventions for mental health professionals (Dobie *et al*, 2016).

We strongly believe that such measures need to be routinely incorporated within psychiatry training programs as part of ‘whole person growth and well-being’ during residency.
